# A qualitative examination of the health workforce needs during climate change disaster response in Pacific Island Countries

**DOI:** 10.1186/1478-4491-12-9

**Published:** 2014-02-12

**Authors:** Michele Rumsey, Stephanie M Fletcher, Jodi Thiessen, Anna Gero, Natasha Kuruppu, John Daly, James Buchan, Juliet Willetts

**Affiliations:** 1 World Health Organization (WHO) Collaborating Centre for Nursing, Midwifery and Health, University of Technology Sydney, P.O. Box 123, Broadway, NSW 2007, Australia; 2 Institute for Sustainable Futures, University of Technology Sydney, P.O. Box 123, Broadway, NSW 2007, Australia

**Keywords:** Climate change, Health workforce governance, Health-care management, Health-care capacity, Competencies, Human resources for health, Workforce development, Emergency, Disaster response, Pacific Islands

## Abstract

**Background:**

There is a growing body of evidence that the impacts of climate change are affecting population health negatively. The Pacific region is particularly vulnerable to climate change; a strong health-care system is required to respond during times of disaster. This paper examines the capacity of the health sector in Pacific Island Countries to adapt to changing disaster response needs, in terms of: (i) health workforce governance, management, policy and involvement; (ii) health-care capacity and skills; and (iii) human resources for health training and workforce development.

**Methods:**

Key stakeholder interviews informed the assessment of the capacity of the health sector and disaster response organizations in Pacific Island Countries to adapt to disaster response needs under a changing climate. The research specifically drew upon and examined the adaptive capacity of individual organizations and the broader system of disaster response in four case study countries (Fiji, Cook Islands, Vanuatu and Samoa).

**Results:**

‘Capacity’ including health-care capacity was one of the objective determinants identified as most significant in influencing the adaptive capacity of disaster response systems in the Pacific. The research identified several elements that could support the adaptive capacity of the health sector such as: inclusive involvement in disaster coordination; policies in place for health workforce coordination; belief in their abilities; and strong donor support. Factors constraining adaptive capacity included: weak coordination of international health personnel; lack of policies to address health worker welfare; limited human resources and material resources; shortages of personnel to deal with psychosocial needs; inadequate skills in field triage and counselling; and limited capacity for training.

**Conclusion:**

Findings from this study can be used to inform the development of human resources for health policies and strategic plans, and to support the development of a coordinated and collaborative approach to disaster response training across the Pacific and other developing contexts. This study also provides an overview of health-care capacity and some of the challenges and strengths that can inform future development work by humanitarian organizations, regional and international donors involved in climate change adaptation, and disaster risk reduction in the Pacific region.

## Background

The intersection between climate change, disasters, health and development is an area of concern for the development community [1-3]. A growing body of evidence links human-induced global warming to an increased number of observed extreme weather events, particularly heat waves and extremes in precipitation, with ‘plausible’ evidence for a link to an increased severe storm potential [4]. Projections for tropical cyclone frequency for the Pacific follow global trends - that is, less frequent, but more intense, tropical cyclones by the end of the 21st century [5]. There is growing political commitment to integrate health considerations into climate change mitigation and adaptation efforts, at different national and regional levels, but these are still limited in the Pacific region [6]. Regional efforts to address climate change and health include the ‘Regional framework for action to protect human health from effects of climate change in the Asia and Pacific region’ and the 2009 Pacific Islands Forum’s call for immediate action to address climate change issues in Pacific Island Countries (PICs) [7]. These efforts were intended to guide regional and national action towards reducing the potential burden of diseases linked to the effects of climate change in the region. The World Health Organization (WHO) regional offices in South East Asia (SEARO) and Western Pacific (WPRO) have continued to engage in and support regional planning activities on climate change and health, resulting in recommendations for action by Member States and WHO secretariat [6]. One of the key priorities identified by regional stakeholders is the need for better understanding of how climate change will impact on health system’s response to emergencies and disasters [8].

The health-care system is directly affected by disasters, and the community is reliant on this system to meet their health needs during and after disasters. It is therefore important that the health sector has adequate capacity to plan for and to respond to post-disaster needs [9,10]. The impact of disasters on population health requires a range of activities to be implemented by the health workforce, demanding various skills and competencies [9]. These include skills in emergency surgery and medical care, rapid diagnosis, disease surveillance, prevention and containment, psychosocial support for affected communities, health education and promotion, creating temporary facilities, conduct monitoring and evaluation, providing medical relief supply management/control and other public health interventions [11-14]. Assessing the vulnerability of the health sector is essential in order to manage future risks, under a changing climate [2,15,16].

The purpose of this research was to assist in identifying factors for enhancing long-term adaptive capacity and thereby inform policy makers and disaster response practitioners on processes required for improved disaster response in the face of climate change. The research defined ‘adaptive capacity’ as the ability of a system to adjust to climate change (including climate variability and extremes), to moderate potential damages, to take advantage of opportunities, or to cope with the consequences [17]. As part of a wider research into the adaptive capacity of the full disaster response system, the capacity of the health sector across the four case study PIC countries was assessed. The concept of ‘adaptive capacity’ is complex and contested, thus, the research team drew on literature across several relevant disciplines to define a range of potential key determinants of adaptive capacity to support exploration of the concept, and are further described in the Methods section. WHO asserts that the capacity of the health-care sector to respond to disasters is a determinant of overall adaptive capacity of human resources for health in disaster response in PICs [18,19]. This paper looks closely at the health-care capacity of the PICs to respond to disaster under a changing and variable climate, based on findings from fieldwork and stakeholder interviews in Australia and four PICs: Cook Islands, Fiji, Samoa and Vanuatu.

## Methods

The research methods are described only briefly here, and may be found in detail elsewhere [20]. In summary, a qualitative research methodology was used, and included desktop reviews, individual and group interviews, and in-country workshops. The research process was guided by a Project Reference Group (PRG) comprising key stakeholders in Australia and the Pacific as a form of structured stakeholder engagement and to guide the research process, outputs and uptake of results. The concept of adaptive capacity was used to identify the factors enabling and constraining individual organizations and the broader system of response [21].

A conceptual framework by Gero et al. [20] guided the research, leading to efforts to both define and understand the disaster response systems, and to identify the most significant determinants affecting adaptive capacity and how these might be influenced to reduce disaster impacts on responding agencies and the wider population. Specific determinants of adaptive capacity were used to assess the DRS, and were drawn from literature that spans Earth System Governance [22], climate change adaptation [23], health resources [1,18,19], resilience in institutions [24] and practice theory [25], several of which are applicable to the health-care setting.

Key determinants of adaptive capacity were identified as being inter-organizational, intra-organizational, objective and subjective. Among these, the intra-organizational - objective determinants of leadership, management and governance structures, technical capacity, tools, methods and approaches, health workforce education, training and continuing competence [18,26-29], human resource for health governance and management systems [18,19], are relevant to this paper.

### Country selection

Four PICs (Cook Islands, Fiji, Samoa and Vanuatu) were investigated in detail to provide more of a regional viewpoint. These countries were selected with the aim to include a variety of geographical settings (including Melanesia and Polynesia), varied policy landscapes (regarding climate change, disaster and health-related policies and plans), a mix of countries with recent significant climate-driven disasters and those in the more distant past (relating to degrees of memory and levels of preparedness), and countries that experience tropical cyclones as an example of a rapid onset disaster. The scope of the study was limited to health-related humanitarian needs and as such researchers were interested in obtaining a mix of countries including high and low human resources for health (HRH) density. Development indicators (for example, varied Human Development Index - see UNDP 2011 [30]) were also used as a criterion for selection to include countries at different stages of development. A full account of the methodology and further background on each country can be found in Gero et al. [20].

### Data collection and analysis

Relevant organizations in each country were identified with the assistance of the PRG, with additional organizations identified through a snow ball sampling technique [31]. Over 90 interviews were undertaken with senior officials from government and non-government organizations (NGOs), UN agencies, donors and regional organizations involved in disaster response from Australia, New Zealand and the four PICs, during May to July 2012. The interview style, developed with the guidance of the PRG, was designed to appreciate the cultural differences and the need for flexibility when interviewing people from various cultural backgrounds. The semi-structured interview guide was informed by the conceptual framework. Examples of health-related questions are provided below as well as the range of organizations interviewed.

Examples of health-related questions:

1. How was the Ministry of Health involved in the coordination of in-country health staff during the disaster? - if/ how did this link in with incoming support?

2. How did incoming Australian or overseas support affect your organization’s ability to respond?

3. How is the health sector involved in the decision-making process regarding requesting external assistance? What role do they play?

4. Does your country have policies in place to coordinate HRH in times of disaster? Are there registration issues for incoming health professionals?

5. How do you think the national system of disaster response will cope if disaster frequency and intensity increases?

Examples of organizations included in interviews:

Australian organizations:

AusAID

The Australian Council for International Development (ACFID)

The Australian Civil Military Centre

Australian Defence Force

Australian Red Cross

NGOs including: Oxfam, World Vision, Save the Children, CARE, Caritas, Plan and RedR, ADRA

Pacific organizations:

National Disaster Management Offices

National Climate Change Offices

Ministries of Health, Environment, Finance, Foreign Affairs (among others)

National Red Cross Societies

Faith-based organizations

NGOs of both local and international origin

Donors (for example, AusAID, New Zealand Aid Programme)

United Nations (UN) agencies

Regional organizations (for example, Secretariat of the Pacific Community (SPC))

Following in-country work, key informant interview transcripts were subjected to an inductive thematic analysis [32], for general patterns and emerging issues from participants’ explanations and descriptions [33]. Interview transcripts were coded for specific themes based on the conceptual framework, including triangulation of data from multiple sources [34] using the qualitative software NVivo 10. Written informed consent was obtained from all participants and only quotes from those who gave consent have been included in this report. This research was approved by the Human Research Ethics Committee of the University of Technology Sydney and by the Ministry of Health in each of the four PIC countries.

## Results and discussion

The findings presented are reflective of the intra-organizational objective determinants of adaptive capacity which are specific to the health sector. The following themes from the analysis of the findings are suggested as being essential for the health-care system, in the four case study PICs, to effectively adapt disaster response in the climate change context. The three thematic areas are:

(i) Health workforce governance, policy and management;

(ii) Health-care capacity and skills;

(iii) Human resources for health training and workforce development.

A summary of key regional health-care capacity issues identified in the four PIC countries are grouped under these three areas and presented in Table 1.

**Table 1:** Summary of key regional health-care capacity issues for disaster response

	**Cook Islands**	**Fiji**	**Samoa**	**Vanuatu**
**Health workforce governance, management and policy**				
**Health-care governance**	No clear guidelines for the coordination of in-coming health personnel. Mechanisms being developed to deal with this.	In-coming health personnel were usually coordinated through the UNOCHA/PHT system.	Existing guidelines for registration of in-coming health professionals, but not always followed.	No clear guidelines for the coordination of in-coming health personnel. Policies were needed to govern this process during disasters.
**Health-care management systems**	Health sector is a key stakeholder in the DRS and well organized and has seen improvements since the clarification of roles and responsibilities.	Health sector is a key stakeholder in the DRS and actively involved in disaster coordination. Health sector coordination functioning well.	Health sector is a key stakeholder in the DRS however internal issues may be affecting the strength of their coordination and involvement.	Health sector is a key stakeholder in the DRS however lacks coordination within the sector and with external partners needs to be improved.
**Health-care policy environment**	Policy in place to guide health workforce. Knowledge of policies affected by high staff turnover rates.	Generic policies and processes in place but need to be more clearly defined for specific disasters.	Clearly defined policies and processes in place, supported by National Development Strategy.	Lack of clear policies and guidelines for health workforce coordination.
**Health-care capacity and skills**				
**Human resources for health capacity**	Clear leadership and strong partnerships with NGOs and donors; health workforce and capacity inadequate particularly in times of disaster.	Strong leadership and external support systems from government and donors; health workforce capacity is stretched especially in times of disaster.	Strong leadership and external support systems from government and donors. Lack of cohesion within health sector limits effective utilisation of resources.	Internal leadership needs strengthening and relies heavily on external support. Limited capacity to respond to disasters against limited HRH and resources.
**Health workforce capacity and skills**	Inadequate capacity to address psychosocial needs.	Inadequate capacity to address psychosocial needs.	Inadequate capacity to address psychosocial needs.	Inadequate capacity to address psychosocial needs.
**Human resources for health training, competencies and workforce development**				
**Health workforce education, training and development**	Some level of disaster training available for health workforce. Desktop and field simulations or training programs annually. Access to training for some levels of staff is an issue. Need for a nursing education Institution in-country.	Disaster training available and included in nursing curriculum. Access to training for some levels of staff an issue.	Nursing and allied health staff actively involved. Task-shifting and multi-tasking encouraged. Low intakes in nursing and medical programs undermining workforce development. Triage and emergency medical care, post-trauma counselling were critical areas for training.	Health workforce actively involved, but needs to improve skills to deal with climate sensitive diseases. Disaster training available and included in nursing curriculum. Additional training for village health workers and traditional birth attendants needed.

DRS = disaster response system; HRH = human resources for health; NGO = non-governmental organization; UNOCHA/PHT = United Nations Office for the Coordination of Humanitarian Affairs/Pacific Humanitarian Team.

Critical aspects of each of the three areas are discussed in more detail in the following sections of the paper.

### Health workforce governance, management and policy

Health workforce governance, policy and management are required for sustained workforce contributions to improved population health outcomes, including HRH capacities to address disasters [18,19]. Governance has been described as a determinant of adaptive capacity of the health sector for disaster response [1,35]. This requires clear policies and a cross-sectoral national coordination or formal mechanisms of governance including health planning, stakeholder coordination, registration and coordination of in-coming overseas health workforce. Management capacity-building policies and structures should be in place and networks and partnerships of relevant committed leaders and stakeholders should be established and under pinned by the relevant operational processes [15,18,19].

#### ***Health-care governance***

For an adaptive policy environment and effective implementation of plans, the health sector requires a suite of governance-related functions, including clear mandates, effective decision-making and response to community-identified strengths [1]. A greater understanding of governance in the health-care context can provide opportunities to strengthen health sector policy and response action to the effects of climate change [1]. WHO defines leadership and governance as the existence of a strategic policy framework as well as effective oversight, coalition building, regulation, attention to system design, and accountability [6]. In the context of this study health governance specifically relates to the set of rules that define the responsibilities of health system actors, how they operate, and how they relate to one another [36]. The interviews as part of this research found two very important health-care management issues: the coordination and management of incoming (overseas) health workforce; and the protection and management of health staff affected by disasters and involved in disaster response.

#### ***Health-care management systems***

One of the key concerns expressed by both Australian and the PIC respondents was the coordination and registration of in-coming international health personnel in times of disasters. This was necessary to ensure timely external assistance, minimize duplication of scarce resources, provide a transparent process, and maximize effectiveness of health personnel. Studies have revealed that these elements are essential for the effectiveness of international disaster responses, and require the development of policies and agreements against agreed criteria as part of disaster preparedness on an international, bilateral and national level [37].

There were different approaches to the coordination of incoming international health personnel in the four PICs. In the Cook Islands, guidelines for the coordination of in-coming health personnel require clarification. In normal times in-coming personnel follow a registration protocol; however, in times of disaster the process was reportedly unclear. This has resulted in a recent review of legislation around international disaster response, calling for stricter regulation in this area [38]. For instance, according to one respondent, the request for international health staff goes through the Disaster Council and the Disaster Committee, who would then advise the Ministry of Health (MOH) of who is coming. However, another government respondent felt that while the requests go through the health ministry, the process can sometimes be informal and can result in fragmented communication, as evidenced by the following quote. “*Informally all requests [for] incoming health workers go to the MOH; [however]it doesn’t always work as it can be an informal process across most sectors, which can lead to lack of sectors talking to each other*”. According to Bremer [37], policy making related to disaster response must be conducted in the disaster planning phase in both receiving and donor countries, since there is little time for policy making and implementation of rules during the acute phase of a disaster. These policies should be carefully developed to guide interventions that are based on commonly agreed upon criteria [37].

However, the absence of policies to guide international HRH in some PICs could be due to the fact that in recent times there has not been a need for international health personnel to assist with disaster response. The infrequency of larger scale disasters impedes the development of an evidence base to inform public health preparedness strategies [39]. One recent example of a disaster requiring overseas health workforce support in the Pacific was during the 2009 tsunami in Samoa [40]. The Pacific Humanitarian Team (PHT) includes, as members all organizations that have a mandate and the capacity to respond to a disaster in the Pacific region. The PHT provides support to government coordination efforts during a disaster response following a request for assistance, and was a key player in the 2009 tsunami response [40]. Both Vanuatu and Fiji respondents indicated that they relied on the PHT/UN Cluster system to assist with provision of health personnel in times of disaster. Samoan respondents indicated that a system was in place for the registration of in-coming health professionals. However, problems have been reported with this process, mainly because some NGOs were either unaware of the need to register or chose to bypass the system. One Samoan respondent suggested that an acceptable approach would be that medical NGOs and volunteers coming from overseas should link directly with the local MOH for a centralized approach in order to avoid duplication of roles. However, there is “*[n]eed for a new fast circuit approach to facilitate the registration and checking of qualifications of health workers at the time of disasters*” (OUM, Samoa).

In trying to address issues with in-coming health personnel, disaster response stakeholders have put forward a recommendation that the relevant Emergency Management Office should work closely with the UN Office for Coordination of Humanitarian Affairs (UNOCHA) and other external organizations to ensure that all personnel are registered and that a proper register of in-coming support is maintained. This will ensure that in-coming personnel are properly screened and facilitate quicker immigration and customs processes [37]. International donors and Australian NGOs indicated that their provision of HRH for humanitarian response is guided by minimum global standards. Ministries of Health in each country should work with external partners to manage registration of incoming health personnel effectively.

The other critical issue of governance involved limited systems for the protection and management of health staff affected by disasters. This was identified as impacting the health workforce’s ability to respond effectively, since they were usually first responders in all countries, and had to conduct disaster initial assessments.

Some international organizsations recognized that disasters impact individual health workers when their families, friends or homes are directly affected, placing additional demands and challenges on health workers [10]. Appropriate support systems would ensure that health workers are cared for and motivated in conducting this essential health function. Efforts and alternative sources that are being investigated to support human resource capacity were described by participants. In Samoa, for example, “*private doctors and nurses are called in to assist in times of disasters*” (Govt. Rep, Samoa).

The pre-existing HRH shortage in the Cook Islands reportedly resulted in existing personnel working extremely long hours. However there was no financial support or benefits for working overtime. Additionally, staff were not insured, so in the event of injury or death in the line of duty there was no redress. Similar issues were identified by respondents from Fiji and Samoa who indicated that health personnel were among the first responders, arriving in the disaster area while it was still relatively unsafe. Since health personnel are usually among the first responders to disasters, there needs to be stronger support systems in place to protect their wellbeing during times of disaster. This does not only have implications for response personnel’s job security, but also for occupational safety and health, insurance and indemnity [41,42]. The provision of adequate pay and adequate indemnity for health-care staff involved in disaster response have been identified as essential motivators for Australian relief personnel [41]. Unfortunately these realities do not translate to their Pacific Islander counterparts due to pre-existing disparities in health-care financing and staff incentives. Efforts should be made to improve conditions for PIC counterpart health workers.

#### ***Health-care policy environment***

Interviews in the four PICs revealed evidence of a mixed policy environment across countries for the coordination of HRH for disaster response. For example, in Fiji a contingency plan for disaster response and coordination of health staff was in place, including legislation to empower key individuals to make decisions in times of disasters. In the Cook Islands, a health disaster policy was in place to provide directions for both on-duty and off-duty staff during disasters, however there was limited knowledge of policies due to high staff turnover rates. The Samoa National Health Service (NHS) has a disaster plan which outlines staff responsibilities, which is disseminated down to the divisional level of the health sector that work closely with communities. The Samoan NHS plan makes provision for the involvement of the private medical practitioners and the Samoa Red Cross Society. Conversely, there was a lack of clear policies and guidelines for health workforce coordination during disasters in Vanuatu. Unfortunately, there was little evidence of strategic planning to ensure that health-care needs were adequately represented in wider planning for climate-related disaster response. This extended to a lack of strategic planning for HRH to meet current and future needs, and may reflect a wider lack of attention to the HRH strategy development process [43]. A good quality strong evidence base is needed for the development of adequate disaster response policies, plans and procedures to guide HRH response. Unfortunately despite a substantial amount of publicly available international literature relating to emergency response planning, the validity and generalizability of these to the Pacific context remains unclear [44].

### Health-care capacity, skills and competencies

The capacity of the health system and its workforce to effectively respond to disasters will be influenced by the magnitude and impact of the disaster as well as the characteristics of the affected system and the availability of adequate resources to respond to needs [10]. Human resources management is essential to any health-care system. The size, composition and distribution of the health-care workforce to meet a particular country’s present and future needs are essential for consideration in the climate driven disaster context [45]. An agile workforce with highly specialized skills is required to mount an immediate and effective response to disasters and humanitarian emergencies [11-14]. Here we describe the human resources capacity, skills, competency, development and training for disaster response in the case study countries.

#### ***Human resources for health capacity***

Climate-driven disasters place added stresses on health-care systems, and where these systems are already understaffed and overstretched, disease burden and health-care needs arising from disasters can exceed HRH response capacity [13,46]. Both Australian and international organizations and PIC respondents have acknowledged that PICs can be easily stretched beyond their HRH capacity in times of disasters, requiring external assistance for surge capacity. One Australian representative alluded to the fact that some countries do not have the capacity to conduct field triage or set up isolation wards, and NGOs often lack a clinical expertise. This is an area where military capabilities are often relied on [47]. However coordination challenges between military, civil society and NGOs can constrain the effectiveness of medical response [47]. The New Zealand Aid Programme also has capacity for HRH support from New Zealand; however offshore field medical care and search capacity was reportedly limited.

There was evidence of collaboration and partnerships with NGOs, community-based organizations and donor organizations to improve HRH capacity for emergency response. Both the WHO and UNICEF regional offices in Fiji reported that Red Cross Society Volunteers and Village Health Workers improve capacity to respond to health emergencies. For example, the Cook Islands Ministry of Health partnered with WHO, Red Cross and communities to respond to a dengue fever outbreak. In other situations, HRH capacity was reportedly managed through intra-island rotation (rural to urban and vice versa). Collaborations and partnerships provide a supportive environment to build adaptive capacity of the health sector and may be an indication that climate change is becoming the impetus to unite organizations and sectors around policy making to address HRH needs of PICs [48]. Other lessons were learned from the Samoan response to the Avianic flu response, where strategic health management resulted in the use of multidisciplinary health teams to mount a rapid and effective response [49]. According to one respondent:

“*We had swine flu before the tsunami so we trained unemployed youth as health assistants in villages so that they can help on the district hospitals during disaster events. The idea was about how these youth can help out in the district hospitals before the formal help arrives (e.g., wound dressing). This training helped when the tsunami hit; we’re trying to apply this approach to up-skill in psychological training. Multi-skilling communities to help themselves partly address the shortage of nurses and medical practitioners in Samoa*” (OUM, Samoa).

Health-care workers in both Fiji and the Cook Islands indicated their willingness to respond to their country’s needs since they have a better understanding of local policies and cultural issues, than external counterparts. There was some levels of hesitancy in requesting health workers from overseas, with some respondents suggesting this as a last resort because “*if they [overseas health workers] come in they have to learn the guidelines and policies of the facility very quickly to be able to work in it*” (MOH-WestDiv, Fiji). The general feeling across PIC health-care interviewees was that despite their limited HRH capacity, the lack of physical resources for emergency response was even more critical. According to some respondents, the limited workforce can efficiently manage available resources for greater effectiveness. Limited finances, emergency medications and other disaster supplies were identified as additional challenges which affected emergency response, especially to outer islands and remote areas.

“*Without funding we can send 8 or 9 [persons] but no medication, because we can charter a flight. With more funding we can take more staff and medicines*” *(MOH, CI).*

The literature indicates that strong health-care systems require significant resources and high levels of preparedness to improve health sector performance and outcomes in disasters [50]. The limited resource capacity in PICs has resulted in heavy reliance on donor funding and bilateral programs. While there is much funding available in the region for climate change and disaster risk reduction programs, very little of this is being channeled into climate change-related health programs and the development of the workforce capacity for disaster response. This has further implications for the management of the limited workforce and their effectiveness in disaster response. The limited focus and allocation of climate-related funding to health in the Pacific reflects the wider approach to global health delivery, where some of the very large donors such as the Gates Foundation or the Global Fund, consider climate change mitigation efforts as a government responsibility and not a part of their core business [48]. Given that limited HRH capacity is a reality in all four PICs, it is unlikely that donor-dependence will change in the immediate future. However, the sustainability of donor support requires assessment in light of global economic recession and emerging competing and other public health priorities in donor countries [51].

#### ***Human resources for health skills and competencies***

Effective disaster response require a combination of the right people with appropriate training, being in the right place at the right time, with sufficient emergency resources [50]. In an ideal situation, the health sector should seek to maximize the functions of the health workforce, retain an efficient mix of staff and skill to improve service which can be applied in disaster response [52]. Disaster situations often require that the focus be changed from routine behaviours to those suited to disaster scenario.

Nursing staff, in all PICs, were actively involved in disaster response, based on their pre-existing roles. The general feeling was that nurses are expected to play a key role in post-disaster leadership and early assessment, providing information to various organizations. The disaster assessments responsibility is delegated to nurses in their capacity as civil servants and often the focal person in health facilities in remote areas. However, while carrying out their responsibilities, their confidence, skills and expertise in this area are often not acknowledged at the planning and management level. Recognition of the vast local knowledge and experience of the health workforce could be an asset to the wider disaster management process, in helping to understand the priority needs of affected communities. Recognition and adequate training of the health workforce would lead to adequate preparation, skills and competencies for effective disaster response [52]. According to one nurse leader in Fiji, “*hospital-based nurses in urban areas need to be orientated to disaster management before going to the outer islands, and training should be arranged regularly to facilitate this*”*.* The need for training based on evidence has been highlighted by the disaster response sector [10].

The limited technical capacity to handle the immediate psychosocial needs post disaster was identified in all PICs, and affected the community, health-care workers and other response personnel. This resulted in heavy reliance on NGOs and the church community to address psychosocial needs. There was agreement across the health sector in all countries that in-country mental health capacity was severely lacking even in normal times. Early psychosocial interventions are required following disasters, especially those resulting in extreme/widespread property damage, trauma and loss of life, and interruptions to livelihood [53]. This is particularly critical in developing settings, where existing HRH shortages, issues affecting social determinants of mental health and wellbeing may be exacerbated by disaster impacts [53,54]. Inter-agency cooperation and coordination are important to ensure appropriate HRH is sourced to provide culturally appropriate mental health response [13,54].

Critical shortages of paramedical staff for field triage were also reported. These critical shortages of technical expertise for emergency response in PICs were reportedly, at times, filled by international HRH. Regardless of the HRH challenges, the research found evidence of high levels of commitment, multi-tasking and flexibility among health workers involved in disaster response.

“*Within the MOH the only directive I received was (to) ‘manage your officers that they are at work,’ … based on our policies we looked to see who can be discharged home; we tried to minimize the people who were within the facility. Because we were stretched, we went onto 12 hour shifts… we would call the nurse and say ‘prepare for a 12-hour shift, bring some extra pants and extra water’. It might sound militant but it helps*” (MOH-WestDiv Fiji).

There was evidence of resilience developed through respondents’ belief in their ability to cope and adapt to climate influences on disasters [24,55]. The long-term impact of the chronic shortage of HRH, especially if there are more frequent and / or severe disaster impacts, may eventually undermine this resilience as a result of limited staff being stretched beyond their capacity and their inability to maintain competencies and skills. The reliance on overseas support for technical expertise areas such as field triaging and mental health is not sustainable and requires urgent attention. There is therefore need for further assessment of how the workforce capacity can be improved in terms of the numbers, skills and competencies for improved disaster response, in light of uncertainties surrounding future pattern of disasters.

### Human resources for health training and workforce development

A properly trained and competent workforce that is aware of and prepared to meet present and future needs is essential for a successful health-care system [45]. The availability of a workforce with specialized skills for disaster response requires adequate training institutions and programs where these skills can be developed and updated [18].

Interviews revealed training institutions for nursing personnel in all three of the four countries; however doctors from across the region were mainly trained in Fiji, with a small cadre being trained in Samoa. Respondents in Fiji and Vanuatu indicated that disaster response was included in their nursing curriculum. Others reported efforts to ensure some level of in-service disaster training including desktop simulations, field simulations or training programs at the country level; including participation in multi-stakeholder field simulations from time to time. Across the four countries, HRH were interested in accessing disaster-specific training. However, one of the challenges to accessing training in the Pacific was the impact of small workforce. One donor indicated that since some organizations only had a few staff, this made it difficult to send people away for training, “*[b]ut the Governments are committed towards training them*” (WHO, Fiji). On the other hand, the challenge was reportedly to ensure that the right individuals were selected to participate in available training. Senior staff were reportedly more likely to be selected for training, but knowledge was not necessarily transferred to frontline or field staff. Samoan respondents indicated that triage and emergency medical care training should be disaster-specific. Disaster response training, including scenario planning is essential to building resilience [24].

Other challenges included the need for training in child psychology and post-trauma counselling in all countries. Additionally, “*the limited numbers of students enrolled into medical and nursing training programs were insufficient to meet the current demands of the country*” (OUM, Samoa); which underscored wider HRH recruitment issues across the region [56].

The issues with training and workforce development for disaster response are reflective of the wider HRH training issues such as low enrolments, shortage of workers, limited succession planning and access to appropriate courses, which are evident across the Pacific region [56]. These limitations can undermine the adaptive capacity of the health workforce for future response, since they affect recruitment, retention and stability of the workforce [57], and long-term resilience [24]. The importance of matching and forecasting the needs, demand and supply of HRH and the provision of training to build resilience and meet the health system needs of individual countries cannot be overemphasized [58]. While examples of adaptiveness including the upgrading and multi-skilling of nurses to address the shortfall in nursing and medical personnel have been reported, this was not representative across countries. There was room for exploring alternative and traditional mechanisms. Health workforce training and development may therefore be one of the most vulnerable aspects of the health-care capacity in PICs, and should be prioritized by national health sectors and donors with a view to building adaptive capacity for disaster response in the context of climate change and future uncertainty.

## Conclusion

There is a growing body of evidence that the impacts of climate change are affecting population health negatively, including those in the Pacific region. Hence, an appropriately trained, qualified and agile health workforce is required to improve response to disasters. This is the first paper that looks specifically at the adaptive capacity of the health sector to disaster response in the climate change context in Pacific Island Countries. The research found several elements that are likely to support or constrain the adaptive capacity of the health-care sector to effectively respond to disasters, in the face of uncertainties presented by climate change.

Elements serving to strengthen the adaptive capacity of the individual country health-care sectors were: widespread involvement of the health sector in disaster response; health workforce beliefs in their own abilities and commitment to respond to their country’s needs even in the face of limited resources; strong support from the NGO and donor community within the region, with Australia and New Zealand providing technical expertise and resources where these were lacking in respective countries.

Elements serving to constrain adaptive capacity included: variable and complex coordination and registration of international health workforce for disaster response; inadequate policies to coordinate HRH for disaster response in some countries and to address the welfare of staff and workers compensation during and following disasters; limited HRH capacity even in times of ‘normality’ means that staff are stretched beyond their capacity; chronic lack of resources and effective health-care provision; severe shortages of technical expertise to meet psychosocial needs of the population, HRH and response personnel across all countries; inadequate skills and competencies in field triage and medical care; limited capacity for training of health workforce specialists in PICs.

This study reveals evidence of resilient elements of HRH in PICs. However several areas of vulnerability need to be addressed through the development of national HRH policies, strategic plans for disaster response, and a coordinated and collaborative approach for disaster response training which may be applicable to other areas across the Pacific Region.

### Further research is recommended

a) The specific training needs of the health workforce to respond in times of disaster given the acknowledgement they work with limited human and material resources.

b) The disaster assessment processes. Currently nurses using their local knowledge are first assessors; however, this is followed by numerous other assessments resulting in duplication, a lack of acknowledgement and information sharing.

c) How the workforce capacity can be improved in terms of the numbers, skills and competencies for improved disaster response, in light of uncertainties surrounding future pattern of disasters.

## Abbreviations

ANGO: Australian Non-Governmental Organization; CI: Cook Islands; HRH: Human resources for health; MOH: Ministry of Health; NGO: Non-Governmental Organization; OUM: Oceania University of Medicine; PIC: Pacific Island Country; UN OCHA: United Nations Office for the Coordination of Humanitarian Affairs; VMGD: Vanuatu Meteorology and Geo-hazard Division; WestDiv: Western Divisional Hospital: Fiji.

## Competing interests

The authors declare that they have no competing interests.

## Authors’ contributions

MR, SF, AG, JT, NK, JW, JB and JD contributed to the conception and design of the study. MR, SF, JT, AG and NK collected the data; MR, SF, AG, JT, NK, JW and JB contributed to the analysis and interpretation of data. MR, SF, JT, AG, JB, JW and JD were involved in drafting the manuscript. All authors were involved in revising the manuscript critically for important intellectual content. All authors have given final approval of the version to be published.
